# The Two Sets of DMSO Respiratory Systems of *Shewanella piezotolerans* WP3 Are Involved in Deep Sea Environmental Adaptation

**DOI:** 10.3389/fmicb.2016.01418

**Published:** 2016-09-07

**Authors:** Lei Xiong, Huahua Jian, Yuxia Zhang, Xiang Xiao

**Affiliations:** ^1^State Key Laboratory of Microbial Metabolism, School of Life Sciences and Biotechnology, Shanghai Jiao Tong UniversityShanghai, China; ^2^State Key Laboratory of Ocean Engineering, School of Naval Architecture, Ocean and Civil Engineering, Shanghai Jiao Tong UniversityShanghai, China

**Keywords:** *Shewanella*, DMSO respiration, high pressure, low temperature, environmental adaptation

## Abstract

Dimethyl sulfoxide (DMSO) is an abundant methylated sulfur compound in deep sea ecosystems. However, the mechanism underlying DMSO-induced reduction in benthic microorganisms is unknown. *Shewanella piezotolerans* WP3, which was isolated from a west Pacific deep sea sediment, can utilize DMSO as the terminal electron acceptor. In this study, two putative *dms* gene clusters [type I (*dmsEFA1B1G1H1*) and type II (*dmsA2B2G2H2*)] were identified in the WP3 genome. Genetic and physiological analyses demonstrated that both *dms* gene clusters were functional and the transcription of both gene clusters was affected by changes in pressure and temperature. Notably, the type I system is essential for WP3 to thrive under *in situ* conditions (4°C/20 MPa), whereas the type II system is more important under high pressure or low temperature conditions (20°C/20 MPa, 4°C/0.1 MPa). Additionally, DMSO-dependent growth conferred by the presence of both *dms* gene clusters was higher than growth conferred by either of the *dms* gene clusters alone. These data collectively suggest that the possession of two sets of DMSO respiratory systems is an adaptive strategy for WP3 survival in deep sea environments. We propose, for the first time, that deep sea microorganisms might be involved in global DMSO/DMS cycling.

## Introduction

Dimethyl sulfoxide (DMSO) concentrations in deep oceanic water are higher than 1.5 nM at depths up to 1,500 m in the equatorial Pacific Ocean and never drop below 1.3 nM at depths up to 4,000 m in the Arabian Sea (Hatton et al., 1996, 1998, 1999). It is thought to be an environmentally significant compound due to the potential role it plays in the biogeochemical cycle of the climatically active trace gas dimethyl sulfide (DMS; Hatton et al., 2005). DMSO can be produced either through the transformation of DMS by both photo-oxidation and bio-oxidation routes or by direct production from marine phytoplankton (Hatton et al., 1996; Simó, 1998; Moran et al., 2012). In addition to its roles in protecting cells against photo-generated oxidants and cryogenic damage, DMSO can also be used as an alternative electron acceptor for energy conservation through microbial dissimilatory reduction

**Table 1:** Strains and plasmids.

Strain or plasmid	Description	Reference or source
***E. coli***		
WM3064	Donor strain for conjugation; *ΔdapA*	Gao et al. (2006)
***S. piezotolerans* WP3**		
WT	Wild type strain	Lab stock
*ΔdmsA1*	*dmsA1* single mutant derived from WT	This study
*ΔdmsA2*	*dmsA2* single mutant derived from WT	This study
*ΔΔdmsA*	*dmsA1* and *dmsA2* double mutant derived from WT	This study
Δ*dmsB1*	*dmsB1* single mutant derived from WP3	This study
Δ*dmsB2*	*dmsB2* single mutant derived from WP3	This study
ΔΔ*dmsB*	*dmsB1* and *dmsB2* double mutant derived from WP3	This study
*ΔΔdmsA-dmsA1-C*	Complemented strain of *ΔΔdmsA* double mutant	This study
*ΔΔdmsA-dmsA2-C*	Complemented strain of *ΔΔdmsA* double mutant	This study
*ΔΔdmsA-*pSW2	*ΔΔdmsA* double mutant containing the empty pSW2 vector as negative control	This study
ΔΔ*dmsB-dmsB1*-C	Complemented strain of *ΔΔdmsB* double mutant	This study
ΔΔ*dmsB-dmsB2*-C	Complemented strain of *ΔΔdmsB* double mutant	This study
ΔΔ*dmsB*-pSW2	*ΔΔdmsB* double mutant containing the empty pSW2 vector as negative control	This study
**Plasmid**		
pSW2	Chloramphenicol resistance, generated from filamentous bacteriophage SW1; used for complementation	Yang et al. (2015)
pSW2-*dmsA1*	pSW2 containing *dmsA1* and the promoter region of *dmsA2*	This study
pSW2-*dmsA2*	pSW2 containing *dmsA2* and its own promoter region	This study
pSW2-*dmsB1*	pSW2 containing *dmsB1* and the promoter region of *dmsB2*	This study
pSW2-*dmsB2*	pSW2 containing *dmsB2* and its own promoter region	This study
pRE112	Chloramphenicol resistance, suicide plasmid with *sacB1* gene as a negative selection marker; used for gene deletion	Lab stock
pRE112-*ΔdmsA1*	pRE112 containing the PCR fragment for deleting *dmsA1*	This study
pRE112-*ΔdmsA2*	pRE112 containing the PCR fragment for deleting *dmsA2*	This study
pRE112-Δ*dmsB1*	pRE112 containing the PCR fragment for deleting *dmsB1*	This study
pRE112-Δ*dmsB2*	pRE112 containing the PCR fragment for deleting *dmsB2*	This study

*RT-PCR, reverse transcription-PCR.*

(Sunda et al., 2002; Asher et al., 2011). Although DMSO acts as the dominant organic sulfur compound in deep oceanic water, the mechanism underlying DMSO bio-reduction by bathypelagic microorganisms is unknown.

Biochemical and genetic analyses of anaerobic DMSO respiration have been performed, particularly in *Escherichia coli* (McCrindle et al., 2005). In *E. coli*, two sets of operons are involved in DMSO respiration: the *dmsABC* operon and the *ynfEFGHI* operon, which is a paralog of the former and is likely to be phenotypically silent (Lubitz and Weiner, 2003). The *dmsABC* operon encodes the three functional proteins DmsA (the molybdopterin cofactor-containing subunit of the DMSO reductase), DmsB (the ion-sulfur subunit), and DmsC (the NapC-like integral membrane anchor). These three subunits constitute a functional DMSO reductase that is anchored to the periplasmic side of the inner membrane by DmsC. The electron released by menaquinol (MQH_2_) oxidation by DmsC is transferred via a series of [4Fe-4S] clusters in DmsB to the catalytic subunit DmsA, which reduces DMSO to DMS (Stanley et al., 2002).

*Shewanella* is a genus of facultative anaerobic, Gram-negative microorganisms that are widely distributed in marine and freshwater environments. The hallmark of *Shewanella* is their ability to utilize a broad range of terminal electron acceptors, which makes them outstanding candidates for potential applications in the bioremediation of pollutants (Nealson and Scott, 2006; Hau and Gralnick, 2007). In *Shewanella* species, the DMSO reduction pathway has been characterized only in *Shewanella oneidensis* MR-1, which was isolated from the sediment of Oneida Lake in New York (Venkateswaran et al., 1999). Two *dms* operons were found in the MR-1 genome, although only one of them mediated DMSO reduction under the tested conditions (Gralnick et al., 2006; Fredrickson et al., 2008).

*Shewanella piezotolerans* WP3 was isolated from a west Pacific deep sea sediment at a depth of 1,914 m (Wang et al., 2004; Xiao et al., 2007). Our previous study demonstrated that WP3 was able to utilize DMSO as a terminal electron acceptor for anaerobic growth (Xiao et al., 2007). However, the precise mechanism of anaerobic DMSO respiration by WP3 is still unknown. In this study, we showed that WP3 contained two *dms* gene clusters (type I and type II), both of which were functional; type I was essential for the ability of WP3 to thrive under *in situ* conditions (4°C/20 MPa) and type II was more important under other extreme conditions (i.e., 20°C/20 MPa or 4°C/0.1 MPa). The possession of two sets of DMSO respiratory systems is suggested to be an adaptive strategy for WP3 to cope with extreme deep sea environments.

## Materials and Methods

### Bacterial Strains and Growth Conditions

The bacterial strains used in this study are listed in **Table 1**. *E. coli* strain WM3064 was routinely grown in Luria Broth medium at 37°C with the addition of 500 μM 2,6-diaminopimelic acid (Sigma-Aldrich, St. Louis, Mo, USA). For aerobic growth, the *S. piezotolerans* WP3 strains were cultured in 2216E broth (Wang et al., 2008; Chen et al., 2011) with minor modifications (5 g l^-1^ tryptone, 1 g l^-1^ yeast extract, and 34 g l^-1^ NaCl) at 20°C in a rotary shaker at 200 rpm. If necessary, chloramphenicol was added to both media (30 μg ml^-1^ for *E. coli* strains and 10 μg ml^-1^ for WP3 strains). For the anaerobic growth assay, the cultivation of WP3 strains was performed in 2216E broth supplemented with 20 mM lactate and 25 mM DMSO (Burns and DiChristina, 2009). Serum bottles each containing 100 ml of fresh medium were prepared anaerobically by flushing with nitrogen gas through the butyl rubber stopper fixed with a metal seal to strip the dissolved oxygen prior to autoclave sterilization. To examine the growth of the WP3 strains at high hydrostatic pressure, each culture was grown to stationary phase in 2216E medium at 1 atm (1 atm = 0.101 MPa) and 20°C in a rotary shaker. The late-log phase cultures were diluted into the same medium to an optical density of 0.3 at 600 nm. Aliquots of the diluted culture (1 ml) were injected into serum bottles containing 100 ml of anaerobic medium through the butyl rubber stopper. After brief shaking, 2.5 ml disposable syringes were used to distribute the culture in 2 ml aliquots. The syringes with needles were stuck into rubber stoppers in a vinyl anaerobic airlock chamber (Coy Laboratory Products Inc., Grass Lake, MI, USA). Then, the syringes were incubated at a hydrostatic pressure of 20 MPa at 4°C or 20°C in stainless steel vessels (Feiyu Science and Technology Exploitation Co., Ltd, Nantong, China) that could be pressurized using water and a hydraulic pump. These systems were equipped with quick-connect fittings for rapid decompression and recompression (Yayanos and Van Boxtel, 1982; Allen et al., 1999).

### Deletion Mutagenesis and Complementation in *S. piezotolerans* WP3

In-frame deletion mutagenesis of *dmsA1* (swp3459) was performed as previously reported (Chen et al., 2011). The primers designed to amplify PCR products for mutagenesis are summarized in Supplementary Table  S1. To construct a *dmsA1* in-frame deletion mutant, two fragments flanking *dmsA1* were amplified by PCR. Fusion PCR products were generated using the amplified PCR fragments as templates with primers swp3459-UF and swp3459-DR. After digestion with the restriction enzymes *Sma*I and *Xba*I, the treated fusion PCR products were ligated into the *Sma*I and *Xba*I sites of the suicide plasmid pRE112 (Edwards et al., 1998), resulting in the mutagenesis vector pRE112-*ΔdmsA1*. This vector was first introduced into *E. coli* WM3064 and then conjugated into WP3-WT (wild type). Positive exconjugants were spread onto marine agar 2216E plates supplemented with 10% (w/v) sucrose. The chloramphenicol-sensitive and sucrose-resistant colonies were screened by PCR for the *dmsA1* deletion. The same strategy was used to construct *ΔdmsA2* single mutants. The double mutant *ΔdmsA1ΔdmsA2* (*ΔΔdmsA*) was constructed by introducing pRE112-*dmsA2* into *ΔdmsA1*. To construct the complemented strain of *ΔdmsA1ΔdmsA2*, the intact *dmsA1* and *dmsA2* gene fragments were amplified from the WT genomic DNA. The resulting PCR products were inserted into the shuttle vector pSW2, which was developed from the filamentous phage SW1 of WP3 (Yang et al., 2015). Transformants *ΔΔdmsA-dmsA1-C* and *ΔΔdmsA-dmsA2-C* were obtained by introducing the recombinant plasmids pSW2-*dmsA1* and pSW2-*dmsA2*, respectively, into the *ΔΔdmsA* strain via conjugation.

### RNA Extraction and Reverse Transcription

Total RNA was extracted from the WP3 strains as previously described (Wang et al., 2009). Briefly, cells in mid-log phase were harvested by centrifugation and treated with the TRI Reagent RNA/DNA/protein isolation kit (Molecular Research Center, Inc., Cincinnati, OH, USA) according to the manufacturer’s instructions. Then, the RNA samples were treated with DNase I (Thermo Fisher, Waltham, MA, USA) to remove residual genomic DNA and quantified using the NanoDrop 2000 spectrophotometer (Thermo Fisher, Waltham, MA, USA), followed by reverse transcription reaction with the RevertAid First Strand cDNA Synthesis Kit (Fermentas, Glen Burnie, MD, USA) to obtain cDNA.

### Real-time PCR (RT-PCR)

Primer pairs (Supplementary Table  S1) for the genes selected for the RT-PCR analysis were designed using the Primer Express 3.0 software (Applied Biosystems, Warrington, UK). Transcription assays were performed using the 7500 System SDS software in reaction mixtures with total volumes of 20 μl containing 10 μl of SYBR Green PCR Master Mix (Applied Biosystems, Warrington, UK), 0.5 μM of each primer, and 1.2 μl of the cDNA template. The amount of target was normalized to the reference gene *swp2079* (Li et al., 2008; Wang et al., 2009). The RT-PCR assays were performed in triplicate for each sample and a mean value and standard deviations were calculated for the relative RNA expression levels.

### DMSO Concentration Determination

Aliquots (1 ml) of the culture recovered at different time points were filtered immediately through 0.22 μm Millex-GP filters (Millipore, Carrigtwohill, Ireland) and stored at -70°C prior to use. The DMSO concentration was determined as previously reported (Cheng et al., 2013). Briefly, the samples were applied for analysis by an Agilent 1200 series high-performance liquid chromatography (HPLC) system (Agilent Technologies, Santa Clara, CA, USA) with a diode-array detector set at 210 nm. DMSO was separated using an Aminex HPX-87H column (Bio-Rad, Hercules, CA, USA) at 50°C using H_2_SO_4_ (5 mM) as the mobile phase at a flow rate of 0.5 ml min^-1^. Commercially available DMSO (Sigma-Aldrich, St. Louis, MO, USA) was used to generate a calibration curve to estimate the DMSO concentration.

## Results

### Determination of the Optimum DMSO Concentration for WP3 Anaerobic Respiration

**FIGURE 1:**
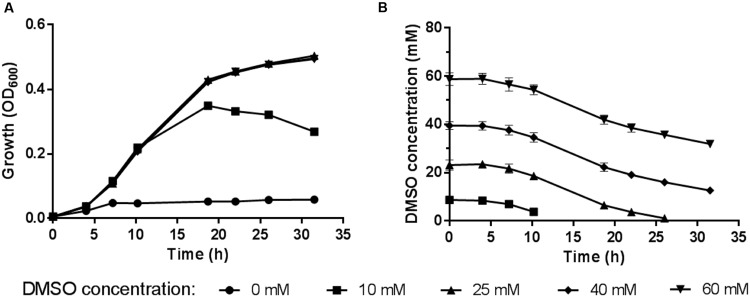
**(A)** Growth of *S. piezotolerans* WP3 in 2216E medium using different DMSO concentrations as the sole electron acceptor. **(B)** Time course of DMSO consumption by *S. piezotolerans* WP3. The data shown represent the results of two independent experiments and the error bars represent standard deviations of the averages of triplicate cultures.

The 2216E medium supplemented with sodium lactate and DMSO was used to study the anaerobic DMSO respiration of WP3. The time course of the DMSO concentration was monitored by assessing the cell density (represented by OD_600_) in the medium (**Figure 1**). The maximum growth was observed at a DMSO concentration of 25 mM, at which concentration the DMSO in the medium was exhausted and the cells were entering stationary phase. We found no significant differences in the cell density and DMSO consumption with a further increase in the DMSO concentration (40 or 60 mM) compared with growth at the 25 mM concentration, suggesting that excess DMSO (40 or 60 mM) had no positive effect on the growth yield. Therefore, the subsequent physiological experiments were performed with DMSO at a concentration of 25 mM.

### Organization of the *dms* Gene Clusters in WP3

**FIGURE 2:**
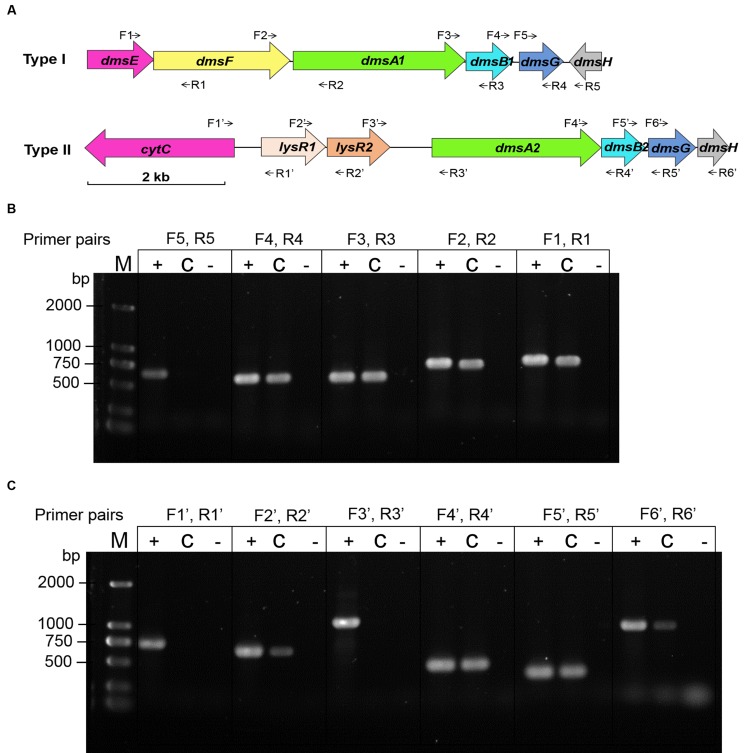
**Co-transcriptional analysis of the type I and type II *dms* gene operons in *S. piezotolerans* WP3. (A)** The operon organization of the two *dms* gene clusters. Arrows indicate primer pairs spanning adjacent genes used for the co-transcription analysis. **(B)** Co-transcriptional analysis of the type I gene operon (*dmsEFA1B1G1H1*). **(C)** Co-transcriptional analysis of the type II gene operon (*dmsA2B2G2H2*). The different templates used for each co-transcription confirmation are presented as follows: +, WP3 genomic DNA (positive control); -, distilled water (negative control); and C, WP3 cDNA. The primer pairs used in each assay are indicated as follows: dmsE-F For/Rev (F1, R1); dmsF-A1 For/Rev (F2, R2); dmsA1-B1 For/Rev (F3, R3); dmsB1-G1 For/Rev (F4, R4); dmsG1-H1 For/Rev (F5, R5); cytC-lysR1 For/Rev (F1′, R1′); lysR1-R2 For/Rev (F2′, R2′); lysR2-dmsA2 For/Rev (F3′, R3′); dmsA2-B2 For/Rev (F4′, R4′); dmsB2-G2 For/Rev (F5′, R5′); and dmsG2-H2 For/Rev (F6′, R6′). The resulting amplicons were analyzed by electrophoresis using 1.0% agarose gels with GelRed staining.

Two sets of *dms* gene clusters [type I (*dmsEFA1B1G1H1* and *swp3461-swp3456*) and type II (*dmsA2B2G2H2* and *swp0724-swp0728*)] were identified according to the WP3 genome annotation (**Figure 2A**). The type I *dms* gene cluster matches the archetypal *dmsEFABGH* organization. However, no *dmsE* or *dmsF* homologous gene was found upstream of *dmsA2B2G2H2* in the type II *dms* gene cluster. Additionally, two partially overlapped genes encoding the LysR family regulator were located directly upstream of *dmsA2*. To investigate whether these two operons were truly polycistronic, reverse transcription PCR was performed to amplify the intergenic regions of neighboring genes. Our data demonstrated that five genes (*dmsEFA1B1G1*) in the type I *dms* gene cluster were co-transcribed, with the exception of *swp3456* (**Figure 2B**). For the type II *dms* gene cluster, four genes (*dmsA2B2G2H2*) were co-transcribed as a single operon (**Figure 2C**).

### Both *dms* Gene Clusters in WP3 Were Functional in DMSO Respiration

**FIGURE 3:**
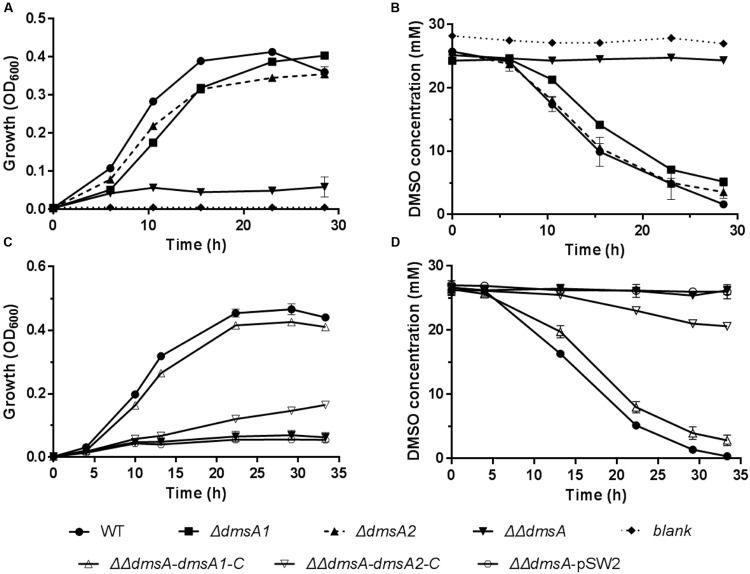
**Growth and corresponding DMSO consumption curves of the WP3 mutants at 20°C/0.1 MPa using DMSO as the sole electron acceptor. (A,B)** Growth and corresponding DMSO consumption curves of WP3 single and double mutants; **(C,D)** Growth and corresponding DMSO consumption curves of WP3 complemented strains. The data shown represent the results of two independent experiments and the error bars represent standard deviations of averages of triplicate cultures.

To test whether both *dms* gene clusters were responsible for DMSO respiration, we initially monitored the transcription of both *dms* gene clusters using quantitative reverse transcription PCR (qRT-PCR). The results showed that both gene clusters were significantly induced under anaerobic DMSO conditions (Supplementary Figure  S1). Because *dmsA* encodes the large catalytic subunit of the DMSO reductase and plays an essential role in DMSO respiration (Trieber et al., 1996), *dmsA* of these two gene clusters was inactivated and in-frame deletion mutants were constructed. The growth assay performed at 20°C/0.1 MPa demonstrated that the single mutants *ΔdmsA1* and *ΔdmsA2* retained the ability to use DMSO for anaerobic growth, whereas the double mutant *ΔΔdmsA* (namely *ΔdmsA1ΔdmsA2*) failed to grow in the same medium (**Figure 3A**) and did not consume DMSO (**Figure 3B**). To confirm that the loss of DMSO-dependent growth of *ΔΔdmsA* was caused by the disruption of *dmsA1* and *dmsA2*, two complemented strains (*ΔΔdmsA-dmsA1-C* and *ΔΔdmsA-dmsA2-C*) were generated. As expected, the introduction of either *dmsA1* or *dmsA2* into *ΔΔdmsA* partially restored the ability of the double mutant to utilize DMSO for anaerobic growth (**Figures 3C,D**). Because *dmsB* encoded the small ion-sulfur subunit of the DMSO reductase, we constructed corresponding in-frame deletion mutants (*ΔdmsB1*, *ΔdmsB2*, and *ΔdmsB1ΔdmsB2*) and complemented strains (*ΔΔdmsB-dmsB1-C* and *ΔΔdmsB-dmsB2-C)*. Similar results were observed in the growth yields and DMSO consumption of these strains (Supplementary Figure  S2). Taken together, these data indicated that both the type I and type II *dms* gene clusters were responsible for DMSO respiration.

### Transcription of the Two *dms* Gene Clusters Was Affected by Pressure and Temperature Changes

**FIGURE 4:**
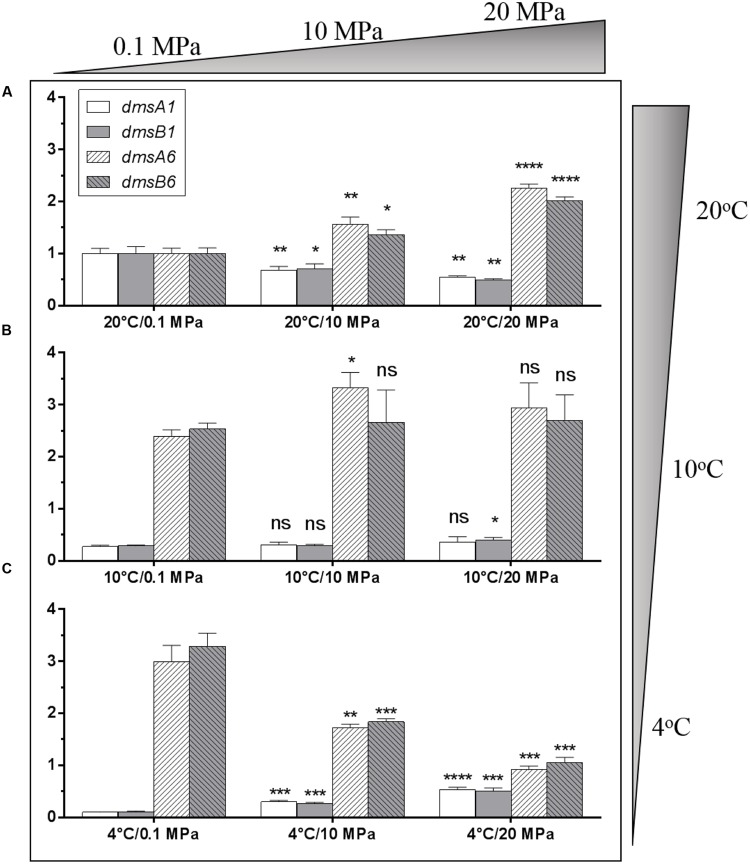
**Transcriptional analysis of two *dms* gene operons in *S. piezotolerans* WP3 under different pressure and temperature conditions. (A)** WP3 grown at 20°C under different pressures; **(B)** WP3 grown at 10°C under different pressures; **(C)** WP3 grown at 4°C under different pressures. The data were normalized to the 20°C/0.1 MPa anaerobic conditions. The data shown represent the results of two independent experiments and the error bars represent standard deviations of averages of triplicate experiments. At each temperature, *P*-value reflects gene transcription differences between atmospheric pressure and high pressure conditions. ^∗^*P* < 0.05; ^∗∗^*P* < 0.01; ^∗∗∗^*P* < 0.001; ^∗∗∗∗^*P* < 0.0001 (Student’s *t*-test); ns, no significant difference.

To evaluate whether these two *dms* gene clusters were involved in DMSO respiration at different temperatures (4 and 20°C) and pressures (0.1 and 20 MPa), relative transcription assays of these two clusters were performed by qRT-PCR. The cells were incubated in anaerobic DMSO medium under different growth conditions for RNA extraction. Compared to the expression levels of the two *dms* gene clusters at 20°C/0.1 MPa, the expression of the type I cluster was significantly down-regulated with pressure increased from atmospheric pressure (0.1 MPa) to high pressure (10 and 20 MPa) conditions, whereas the expression of type II cluster was significantly up-regulated under the same conditions (**Figure 4A**). In contrast, the expression profiles of two *dms* gene clusters at 10°C were generally similar regardless of pressure changes (**Figure 4B**). Interestingly, the expression profiles of type I *dms* clusters at 4°C were significantly induced with pressure increased from atmospheric pressure to high pressure (10 and 20 MPa) conditions, whereas the expression profiles of type II *dms* clusters were greatly reduced under the same conditions (**Figure 4C**). Collectively, these data suggested that the transcription of the two *dms* clusters was regulated in response to pressure and temperature changes.

### The Contribution of the Two *dms* Gene Clusters to WP3 Growth was Influenced by Pressure and Temperature

**FIGURE 5:**
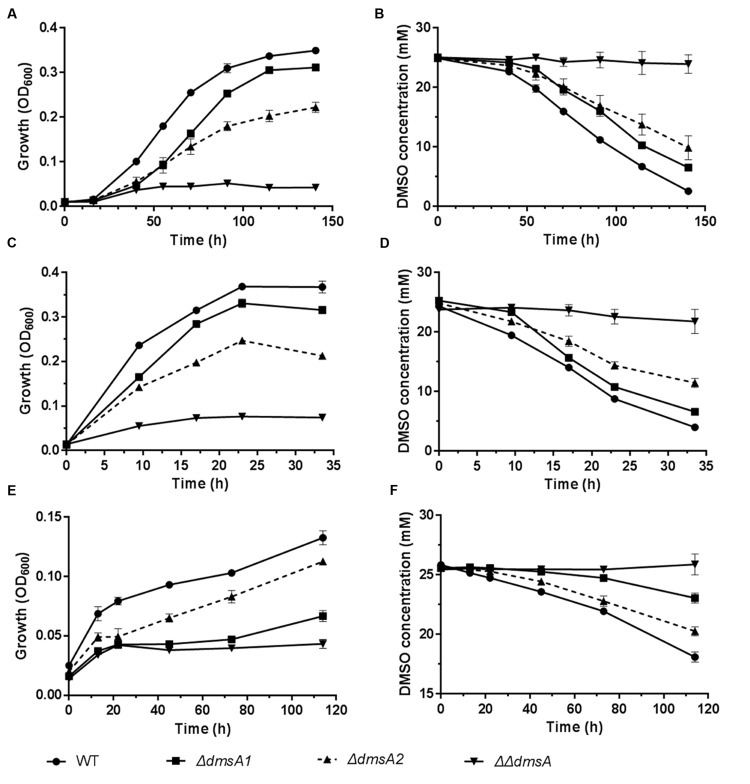
**Growth and corresponding DMSO consumption curves of WP3 mutants at different temperatures and pressures with DMSO as the sole electron acceptor. (A,B)** 4°C and 0.1 MPa; **(C,D)** 20°C and 20 MPa; **(E,F)** 4°C and 20 MPa. The data shown represent the results of two independent experiments and the error bars represent standard deviations of averages of triplicate cultures.

To investigate whether the two *dms* gene clusters were involved in deep sea environmental adaptation, a growth assay was performed at different pressures and temperatures. As predicted, *ΔdmsA1* exhibited a higher growth yield than *ΔdmsA2* when the growth temperature decreased from 20 to 4°C or the pressure increased from 0.1 to 20 MPa (**Figures 5A,C**), which indicated that the type II *dms* operon was more important than the type I operon at low temperature or high pressure. Additionally, culture media collected at different time points were filtered and then analyzed to assess DMSO consumption by these strains. The HPLC results indicated that the *ΔdmsA1* mutant exhibited higher DMSO consumption (**Figures 5B,D**) than *ΔdmsA2* at 4°C/0.1 MPa or 20°C/20 MPa.

Interestingly, when these strains were incubated at 4°C/20 MPa, the growth of *ΔdmsA2* appeared to be higher than *ΔdmsA1* (**Figure 5E**), which was consistent with the more efficient DMSO consumption by *ΔdmsA2* in the medium (**Figure 5F**). Additionally, our results showed that the DMSO-dependent growth yield conferred by the presence of the two *dms* operons was higher than the growth yield conferred by either of the two *dms* operons alone, indicating that the presence of both *dms* operons was essential for the DMSO-dependent growth of WP3 under *in situ* deep sea conditions.

## Discussion

DMSO and DMS are interchangeable substrates in marine environments through the bio-reduction and bio-oxidation pathways (Bürgmann et al., 2007; Muyzer and Stams, 2008; Rellinger et al., 2009; Spiese et al., 2009). As a volatile anti-greenhouse gas, DMS plays an important climatic role in cloud nucleation and hence may influence the global temperature (Charlson et al., 1987; Andreae, 1990; Curson et al., 2011). Previous study demonstrated that DMSO as a substantial sink or source for DMS (Hatton et al., 1996). The formation of DMSO would therefore lead to the removal of DMS from sea water, effectively limiting the quantity of DMS available for releasing into the atmosphere (Hatton et al., 2005). Although DMSO has been detected in abyssal ocean water (Hatton et al., 1999), few data are available on the turnover mechanisms of DMSO in deep sea environments. In this study, our results demonstrate a previously unrecognized role for biotic DMSO reduction as an important pathway of DMS production in the deep sea environment.

In the abyssal ocean, metabolic energy is harnessed from the coupling of redox reactions (Orcutt et al., 2011). Microorganisms exploit the available chemical energy by developing strategies to overcome the activation energy of reaction. The metabolic activities of microorganisms in such environment depend on the availability and speciation of electron donors and acceptors (Froelich et al., 1979). As for DMSO, the *E*_0_′ value for its reduction (+160 mV) lies between those for fumarate/succinate (+33 mV) and nitrate/nitrite (+433 mV; Thauer et al., 1977; Wood, 1981), and thus the coupling of DMSO reduction to ATP synthesis presents no energetic difficulty. Accordingly, some culturable bacteria isolated from marine environments have also shown the ability to grow by anaerobic DMSO respiration (Jonkers et al., 1996; Süß et al., 2008). Although sulfate is the most abundant oxidized sulfur species in the dark ocean (Orcutt et al., 2011), the reduction of sulfate yields less energy than that of DMSO, and DMSO could therefore be consumed preferentially.

In *Shewanella* species, the DMSO reduction pathway was first characterized in *S. oneidensis* MR-1, which was a strain isolated from the sediment of Oneida Lake in New York (Venkateswaran et al., 1999). Two *dms* operons were found in the MR-1 genome; however, only one *dms* operon mediated DMSO reduction under the tested conditions (Gralnick et al., 2006). In contrast to the results in MR-1, both *dms* gene operons in WP3 were functional (**Figure 3**). Moreover, our results demonstrated that the presence of both operons was essential for the maximum growth of WP3 using DMSO as the sole electron acceptor (**Figure 5**). Type I matches the archetypal *dmsEFABGH* organization, which enables the DMSO reductase to localize to the outer membrane of *Shewanella* and allows the cells to respire DMSO more efficiently (Gralnick et al., 2006). Additionally, type I plays a dominant role in the DMSO-dependent growth of WP3 under *in situ* deep sea conditions (4°C/20 MPa). Type II is more similar to *E. coli* (*dmsABC* organization; Stanley et al., 2002) and may locate in the periplasmic space. The physiological analysis indicated that type II was more important for DMSO-dependent growth than type I under some stressful conditions (i.e., 20°C/20 MPa and 4°C/0.1 MPa).

To evaluate the impact of the two DMSO respiration systems on growth, we calculated the DMSO consumption of the WP3 mutants by standardizing the growth yield to the same cell density (1 OD unit ≈ 1.5 × 10^8^ cells; Supplementary Figure  S3). Our data showed that WT (type I and type II together) and *ΔdmsA1* (type II alone) required less DMSO than *ΔdmsA2* (type I alone) to reach the same growth yield when incubated at 20°C/0.1 MPa, 4°C/0.1 MPa, or 20°C/20 MPa, indicating that type II was a more efficient DMSO respiration system at atmospheric pressure or 20°C. However, when the WP3 mutants were incubated at 4°C/20 MPa, *ΔdmsA2* (type I alone) inversely required less DMSO than *ΔdmsA1* (type II alone), indicating that type I was more efficient than type II under the 4°C/20 MPa *in situ* condition. Collectively, our results suggest that the possession of multiple systems with similar functions might be an adaptive strategy for bacteria to cope with extreme deep sea environments. Similar adaptive mechanisms can also be found in other deep sea-derived *Shewanella* strains. For example, *Shewanella putrefaciens* W3-18-1 (Sediment; under 630 m of oxic water) has been identified to contain two functional *nap* gene clusters (*nap-α* and *nap-β*), and the nitrate respiration in W3-18-1 starts earlier than that in MR-1 (with *nap-β* only) under microoxic conditions (Qiu et al., 2013). In another *Shewanella* strain, *Shewanella violacea* DSS12 (Sediment; 5,110 m), it possesses more terminal oxidases with different affinities for oxygen and less terminal reductases, implying that DSS12 has undergone respiratory adaptation to aerobiosis in the upper layer of deep sea sediments (Aono et al., 2010).

The physiological results concerning the possession of two DMSO respiration systems in WP3 are quite different from those of the two NAP systems in WP3. Although two nitrate respiration systems (*nap*-α and *nap*-β) were identified in the WP3 genome, further studies demonstrated that WP3 possessing *nap*-α alone was more competitive than the strain possessing both *nap*-α and *nap*-β during nitrate-dependent growth. Thus containing a single *nap*-α system should be the evolutionary direction for *Shewanella* to thrive at low temperature (Chen et al., 2011). By combining these results, we conclude that WP3 can utilize different strategies to evolve its different anaerobic respiration systems in response to deep sea environments.

The effects of pressure on the respiratory systems has been well studied in *S. violacea* DSS12. Although very few terminal reductases for anaerobic respiration being identified in DSS12 genome, it processes at least five sets of putative terminal oxidases for aerobic respiration (Aono et al., 2010), in which the expression of cytochrome *bd* (quinol oxidase) was enhanced under high pressure (Tamegai et al., 2005). In addition, the *d*-type terminal oxidase of DSS12 was identified to be pressure-resistant and functional under high pressure conditions (Chikuma et al., 2007). Further research demonstrated that the piezotolerance of the *Shewanella* terminal oxidase activity perhaps depends on the intrinsic properties of the enzymes themselves but not their membrane lipid composition (Tamegai et al., 2011). In contrast, the piezophilic property of terminal oxidases in *Photobacterium profundum* SS9 (amphipod; 2,500 m) seems to be different. For one thing, the cytochrome content of *P. profundum* SS9 was not affected by altered pressure during growth; for another, genes encoding cytochromes showed no significant change in expression under different pressures during growth of *P. profundum* SS9. However, the piezotolerance of *P. profundum* SS9 terminal oxidase activity could also be observed using cells grown under higher pressures (Tamegai et al., 2012). Collectively, piezophiles from distinct genus may choose different strategies in adaptating to high pressure.

More recently, the respective effects of temperature and pressure on Fe(III) reduction rates (FeRRs) and viability have been investigated in deep sea bacterium *Shewanella profunda* LT13a (sediment; 4,500 m; Picard et al., 2015). Results showed that FeRR increased linearly with temperature between 4 and 37°C, while the highest FeRR was observed between 10 and 40 MPa and then slightly decreased with increasing pressure (40–110 MPa), indicating that the respiratory chain was not immediately affected by pressure (Picard et al., 2015). Under high pressure conditions, the significantly increased energy demand for cell maintenance of *S. profunda* LT13a may account for its decreased viability (Picard et al., 2015). Here we observed that DMSO-dependent growth can occur when WP3 is incubated in 4°C/20 MPa conditions (**Figure 5E**), suggesting that the energy demand of WP3 could still be fulfilled under *in situ* conditions with DMSO as the sole electron acceptor.

Because the transcription profiles of these two *dms* gene clusters vary significantly in response to pressure or temperature changes (**Figure 4**), it is important to elucidate the mechanism by which *dms* gene clusters are regulated. Previously, a mutant lacking the *fur* gene (encoding the ferric uptake regulator) was found to be severely deficient in DMSO respiration in WP3 (Yang et al., 2013), indicating that Fur was the *dms* gene cluster regulator. Moreover, cAMP receptor protein (CRP) was also found to directly regulate the expression of the *dms* gene cluster in *S. oneidensis* MR-1 (Zhou et al., 2013). We predicted the transcriptional regulation motifs of both *dms* gene clusters in WP3 and found similar CRP binding motifs (data not shown). Taken together, the presence of the CRP and Fur binding motifs might be an alternative regulatory strategy for WP3 to cope with pressure or temperature changes in a subtle regulatory mechanism. Investigation into this mechanism is currently underway and will be described as part of a separate report.

## Conclusion

We have shown that two *dms* operons found in *S. piezotolerans* WP3 are functional in DMSO respiration. Both *dms* gene clusters are essential for the maximum growth of WP3 under low temperature and high pressure conditions; the type I system is essential for the ability of WP3 to thrive under *in situ* conditions (4°C/20 MPa), whereas the type II system is more important under other stressful conditions (i.e., 4°C/0.1 MPa and 20°C/20 MPa). Based on these data, we propose that multiple copies of *dms* gene clusters may confer a competitive advantage to the ability of WP3 to thrive under a broader range of environmental conditions. Thus, deep sea microorganism-mediated DMSO reduction may contribute to the global DMS cycle, which has been neglected in previous studies.

## Author Contributions

LX, HJ, and XX designed the study, analyzed the data. LX and YZ participated in the experiments. LX and HJ wrote the manuscript and XX critically reviewed the manuscript. All authors read and approved the final manuscript.

## Conflict of Interest Statement

The authors declare that the research was conducted in the absence of any commercial or financial relationships that could be construed as a potential conflict of interest.
