# Supplementary material for: The Two Sets of DMSO Respiratory Systems of Shewanella piezotolerans WP3 Are Involved in Deep Sea Environmental Adaptation
Source: Front Microbiol. 2016 Sep 7;7:1418. doi: 10.3389/fmicb.2016.01418 (PMC5013071; doi:10.3389/fmicb.2016.01418)
Supplement: Supplementary file 1 [file Data_Sheet_1.DOCX]

Supplementary Material

**The two sets of DMSO respiratory systems of *Shewanella piezotolerans* WP3 are involved in deep sea environmental adaptation**

**Lei Xiong^1^, Huahua Jian^1^, Yuxia Zhang^1^, Xiang Xiao^1,2,^**^*^

* **Correspondence:** Xiang Xiao: Email: [xoxiang@sjtu.edu.cn](mailto:xoxiang@sjtu.edu.cn)

# Supplementary Figures and Tables

## Supplementary Figures


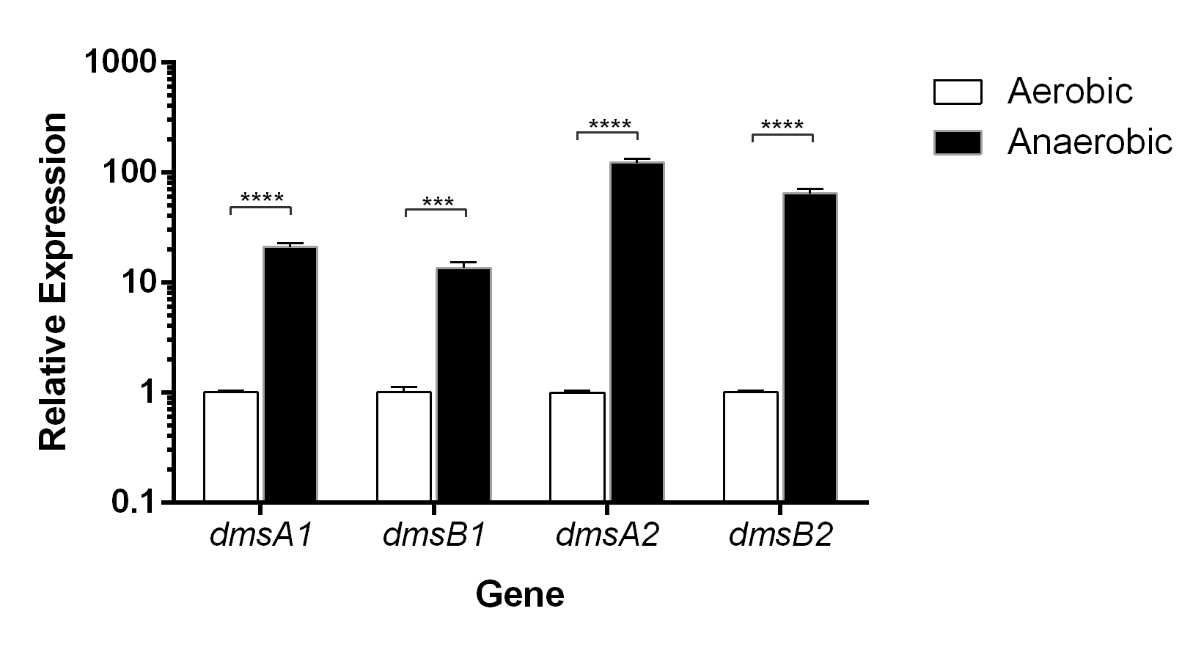


**Figure S1.** Comparison of the transcription of two *dms* gene clusters in *S. piezotolerans* WP3 grown at 20°C under aerobic or anaerobic DMSO conditions. The expression of each gene was normalized to the reference gene *swp2079* and the error bars represent standard deviations of averages of triplicate experiments. The data were analyzed by Student’s *t* test. ***, P<0.001; ****, P<0.0001.


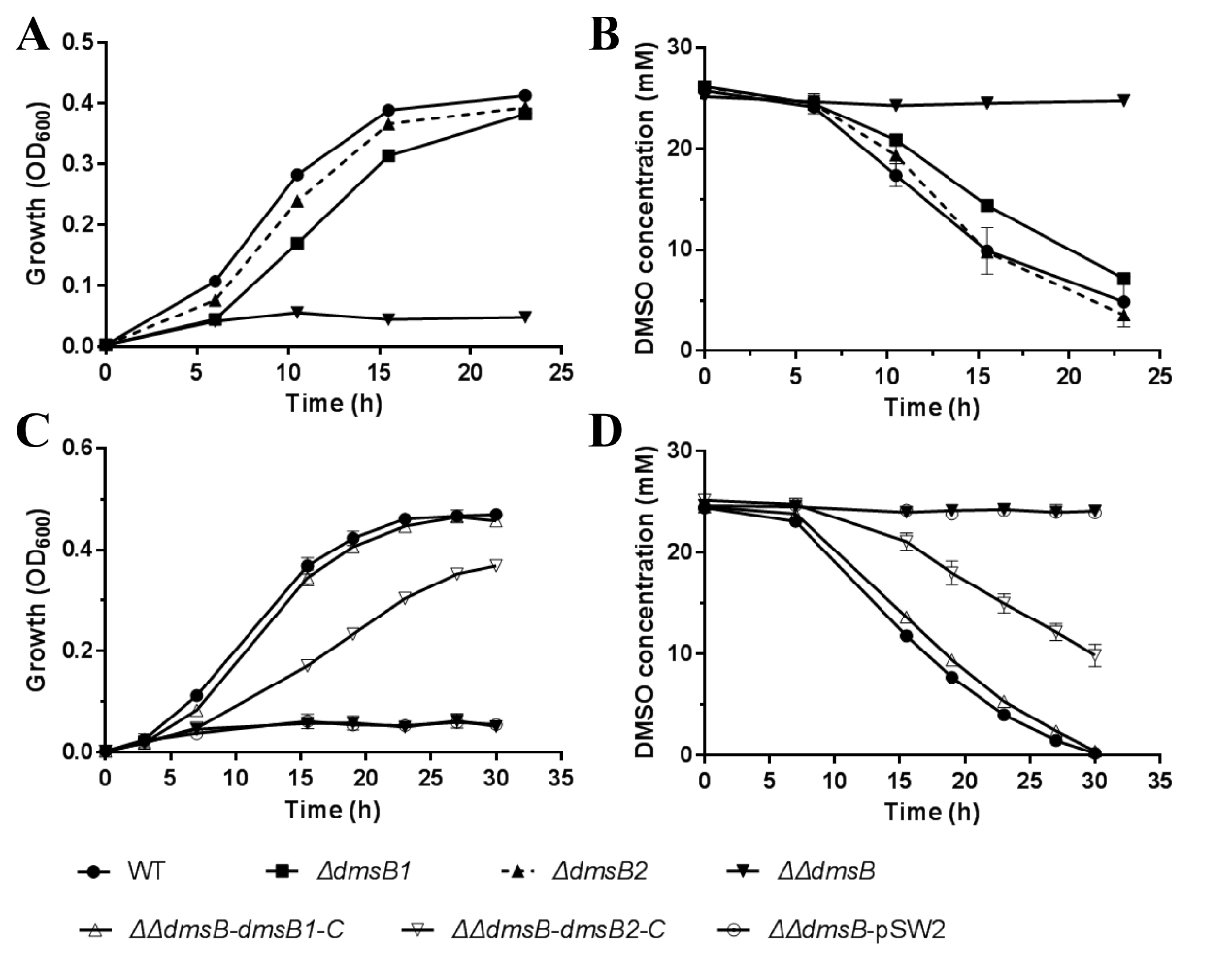


**Figure S2**. Growth and corresponding DMSO consumption curves of the WP3 mutants grown at 20°C/0.1 MPa using DMSO as the sole electron acceptor. The data shown represent the results of two independent experiments and the error bars represent standard deviations of averages of triplicate cultures.


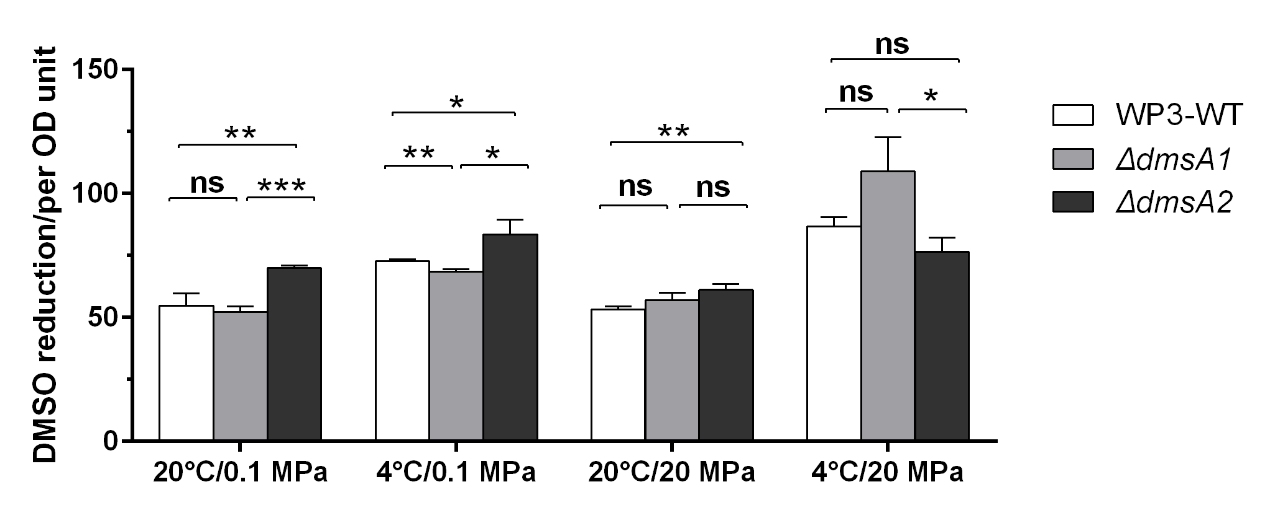


**Figure S3**. Comparison of total DMSO consumption per OD unit of the WP3 mutants under different pressure and temperature conditions. The error bars represent standard deviations of averages of triplicate cultures. The data were analyzed by Student’s *t* test. *, P<0.05; **, P<0.01; ***, P<0.001; ns, no significant difference.

## Supplementary Tables

**Table S1 Primers used in this study.**

| Primers | Primer sequence (5’-3’) | | | Description | |
| --- | --- | --- | --- | --- | --- |
| Mutation use |  | |  | | |
| swp0724-UF | TTGAGCTCCGAGGTAACGCAAAATGAAAAGAC | | Deleting *dmsA2* | |  |
| swp0724-UR | CCTTTAGTAGTAGTACGCGCCAGTGCCATAAA | | Deleting *dmsA2* | | |
| swp0724-DF | GTACTACTACTAAAGGCCGCACCCACTCA | | Deleting *dmsA2* | | |
| swp0724-DR | TTGCATGCGAAACTGCCACCAACAAACTCATA | | Deleting *dmsA2* | | |
| swp0725-UF | TAGAGCTCATGTCGATACGGATTACCCACTG | | Deleting *dmsB2* | | |
| swp0725-UR | TTTGCCA TATAAACGCGACGCTGAATAATACC | | Deleting *dmsB2* | | |
| swp0725-DF | GCGTTTATATGGCAAAGGCGATACTCACC | | Deleting *dmsB2* | | |
| swp0725-DR | AAGCATGCGCTCTGTTAATTTTTCGTCGTTCC | | Deleting *dmsB2* | | |
| swp3459-UF | TACCCGGGTGTTATCTTGCGGGTTGCTATTGT | | Deleting *dmsA1* | | |
| swp3459-UR | TATACCGCTGCCGTTAAGGTTCGTATCACTCC | | Deleting *dmsA1* | | |
| swp3459-DF | TAACGGCAGCGGTATAACATTGGCATCATCAG | | Deleting *dmsA1* | | |
| swp3459-DR | ATTCTAGAAGTAATACCGGAGTCAGCCCTTCT | | Deleting *dmsA1* | | |
| swp3458-UF | AAGGTACCCGGCCTCTGGATGACGAATAAG | Deleting *dmsB1* | | | |
| swp3458-UR | GTGAATGGAGTACGGCGACGGCGATGGA | Deleting *dmsB1* | | | |
| swp3458-DF | GCCGTACTCCATTCACCGCCACCGTATTCAT | Deleting *dmsB1* | | | |
| swp3458-DR | AAGAGCTCTGCCGTTAAGGTTCGTATCACTCC | Deleting *dmsB1* | | | |
|  |  | |  | | |
| Complementation use |  | |  | | |
| Pro-F for dmsA1 | GGTTCTCGAGAGTTGTATCTCACAGTCGTA | | *dmsA1* Promoter | | |
| Pro-R for dmsA1 | CGTTCCATTTTGCGTTACCTCGACATT | | *dmsA1* Promoter | | |
| dmsA1-F | AACGCAAAATGGAACGTAGAAGTTTCTTA | | *dmsA1* amplification | | |
| dmsA1-R | AACTGGATCCTTAAATCTTTTTAATTGCAACACGG | | *dmsA1* amplification | | |
| (Pro+dmsA2)F | GGTTCTCGAGAGTTGTATCTCACAGTCGTA | | Amplifying *dmsA2* and its promoter | | |
| (Pro+dmsA2)R | ATTAGGATCCCCCCTACTTAGCCTTAACCTG | | Amplifying *dmsA2* and its promoter | | |
| Pro-F for dmsB1 | GGTTCTCGAGAGTTGTATCTCACAGTCGTA | | *dmsB1* promoter | | |
| Pro-R for dmsB1 | GTCATCATTTTGCGTTACCTCGACATT | | *dmsB1* promoter | | |
| dmsB1-F | AACGCAAAATGATGACTGAACCAACTC | | *dmsB1* amplification | | |
| dmsB1-R | AGACACGCGTCACCTTTAACACGACGC | | *dmsB1* amplification | | |
| Pro-F for dmsB2 | GGTTCTCGAGAGTTGTATCTCACAGTCGTA | | *dmsB2* promoter | | |
| Pro-R for dmsB2 | TTAGTCATTTTGCGTTACCTCGACATT | | *dmsB2* promoter | | |
| dmsB2-F | AACGCAAAATGACTAATTTAATTCAAAC | | *dmsB2* amplification | | |
| dmsB2-R | ACTCACGCGTTTTTTACCTCACATCAAGT | | *dmsB2* amplification | | |
|  |  | |  | | |
| RT-PCR use |  | |  | | |
| dmsA1 For | AGGCTGTAATTCTAGCTCTGATGATG | | RT-PCR | | |
| dmsA1 Rev | AAGCACGATGACCAGGTTACCT | | RT-PCR | | |
| dmsB1 For | GAAGACATTTGTATCGGTTGTGAAA | | RT-PCR | | |
| dmsB1 Rev | GCGTTCACGGTCAATTTGC | | RT-PCR | | |
| dmsA2 For | GATGACAAATGTATCGGCTGTAATATG | | RT-PCR | | |
| dmsA2 Rev | TTTTTACGCTCAGTATCCATTTGC | | RT-PCR | | |
| dmsB2 For | CACTGCAGTTGGTGGGATACC | | RT-PCR | | |
| dmsB2 Rev | CGTAGCCATGGCACATTATGA | | RT-PCR | | |
| swp2079 For | TTAAGGCAATGGAAGCTGCAT | | Reference gene | | |
| swp2079 Rev | CGTCTTTACCCGTTAATGATACGA | |  |  |  |
|  |  | |  | | |
| Co-transcription assay |  | |  | | |
| cytC-lysR1 For | CATTCTTGCCATCTTCACCATCTG | | F1’ | | |
| cytC-lysR1 Rev | GCTTCGCTCAAATCCCTGCTGTT | | R1’ | | |
| lysR1-R2 For | TAGAGGATATTACCGACCCT | | F2’ | | |
| lysR1-R2 Rev | CTCAATAGAGCGACTACAGG | | R2’ | | |
| lysR2-dmsA2 For | GGTAGCGCAGCGAGTAGTTTGGTT | | F3’ | | |
| lysR2-dmsA2 Rev | CGTAGGGAGCCGTTTCATCAGG | | R3’ | | |
| dmsA2-B2 For | GGCGATATGACCCAAGTGACGAAC | | F4’ | | |
| dmsA2-B2 Rev | CGCGACGCTGAATAATACCATCTG | | R4’ | | |
| dmsB2-G2 For | ATTTTTCTGTGACCAAGCCTAACC | | F5’ | | |
| dmsB2-G2 Rev | TGATCTCATTTTGCGCCAGTAAG | | R5’ | | |
| dmsG2-H2 For | TAAACTTACTGGCGCAAAATGAGA | | F6’ | | |
| dmsG2-H2 Rev | GCGGTTAGCTAGGGACACAATC | | R6’ | | |
| dmsG1-H1 For | TAAGCGTCAATCTAGCCGTAACAT | | F5 | | |
| dmsG1-H1 Rev | AACCGAGATCAAAATAGCACAGGA | | R5 | | |
| dmsB1-G1 For | ATCAAGTGGCTGCGGGTAATCAAC | | F4 | | |
| dmsB1-G1 Rev | CGGCAGGTAGTGGCGAAGGTAG | | R4 | | |
| dmsA1-B1 For | TGTGCATCGCCCCCGTAGG | | F3 | | |
| dmsA1-B1 Rev | GCTCAATTGCCGTTAAGGTTCGTA | | R3 | | |
| dmsF-A1 For | TCAGTTTGTTGCCGATGGTTTTAG | | F2 | | |
| dmsF-A1 Rev | CCTTCTGGTGCGGGTTCTTACG | | R2 | | |
| dmsE-F For | CTGCGTCAATGCCGAAAAGTCA | | F1 | | |
| dmsE-F Rev | CACGTGTGCCGCAATTATGTCAG | | R1 | | |

Abbreviation: RT-PCR, reverse transcription-PCR. The restriction sites included in the PCR primers are underlined.
